# Model-independent recovery of interfacial structure from multi-contrast neutron reflectivity data

**DOI:** 10.1107/S1600576719003534

**Published:** 2019-04-30

**Authors:** Alexandros Koutsioubas

**Affiliations:** a Jülich Centre for Neutron Science (JCNS) at Heinz Maier-Leibnitz Zentrum (MLZ), Forschungszentrum Jülich GmbH, Lichtenbergstrasse 1, 85748 Garching, Germany

**Keywords:** neutron reflectivity, structure recovery, soft interfaces, data fitting

## Abstract

Neutron specular reflectivity at soft interfaces provides sub-nanometre information concerning the molecular distribution of thin films, while the application of contrast variation can highlight the scattering from different parts of the system and lead to an overall reduction in fitting ambiguity. Traditional modelling approaches involve the construction of a trial scattering length density profile based on initial speculation and the subsequent refinement of its parameters through minimization of the discrepancy between the calculated and measured reflectivity. In practice this might produce an artificial bias towards specific sets of solutions. On the other hand, direct inversion of reflectivity data, despite its ability to provide a unique solution, is subject to limitations and experimental complications. Presented here is an integrated indirect Fourier transform/simulated annealing method that, when applied to multiple solvent contrast reflectivity data and within the limits of finite spatial resolution, leads to reliable reconstructions of the interfacial structure without the need for any *a priori* assumptions. The generality of the method permits its straightforward application in common experimental contrast-variation investigations at the solid/liquid and air/liquid interface.

## Abstract 2

An indirect Fourier transform/simulated annealing method exploits the information content of multiple solvent contrast neutron reflectivity data and permits the model-independent recovery of interfacial structure at the air/liquid and solid/liquid interface.

## 1. Introduction   

Specular neutron reflectometry constitutes an established experimental technique for the investigation of the structure of interfaces at the sub-nanometre scale (Penfold & Thomas, 1990 ▸). In particular, in the case of ‘soft’ air/liquid and solid/liquid interfaces involving polymers, lipids, surfactants and bio-molecules the technique has witnessed increased use in the past decade (Braun *et al.*, 2017 ▸; Junghans *et al.*, 2015 ▸; Penfold & Thomas, 2014 ▸; Fragneto, 2012 ▸; Wacklin, 2010 ▸; Nylander *et al.*, 2008 ▸; Sferrazza *et al.*, 2000 ▸), a tendency that is also boosted by advances in sample environment and instrumentation at neutron sources around the world. The essence of a reflectivity experiment consists of registering specular reflectivity *R* as a function of momentum transfer *q* = 4πsinθ/λ where θ is the incidence angle and λ is the wavelength of the incident radiation. Since only the amplitude of the reflected neutron wavefunctions is measured during an experiment, the associated phase information is lost (phase problem), leading to complications regarding unambiguous data interpretation.

In the analysis of neutron reflectivity data, one is faced with the problem of obtaining a reliable scattering length density (SLD) profile of the interface which is physically meaningful and unique. (The SLD profile is defined as the number-density-weighted nanometre-scale average of the scattering lengths of the film’s atomic constituents.) Traditionally, the vast majority of published studies have relied on model fitting for the analysis of reflectivity data, where, on the basis of previous knowledge and intuition, the system is represented as a number of stratified slabs with various parameters (SLD, thickness, roughness) left to be found by conventional least-squares methods (Nelson, 2006 ▸; Gerelli, 2016*a* ▸,*b* ▸). Success in recovering the SLD profile from a single reflectivity measurement depends on the available *a priori* information and on the validity of the initially built model. It has been shown in many studies (Fragneto *et al.*, 1995 ▸; Braun *et al.*, 2017 ▸; Wacklin, 2010 ▸) that the concurrent fitting of multiple contrast data, which can be acquired in neutron reflectivity experiments by the manipulation of solvent SLD, can decisively aid in obtaining an objective final solution.

In a series of pioneering studies, Marjkrzak and co-workers (Majkrzak & Berk, 1998 ▸; Majkrzak *et al.*, 2003 ▸, 2000 ▸) developed exact methods for determining the phase in neutron reflectivity measurements, thus making it possible to extract the SLD profile via direct inversion. These methods rely either on the use of reference layers or on the controlled variation of the contrast of the fronting or backing medium. Preparation of substrates with reference layers adds complexity to the experimental investigation, requires extensive pre-characterization of the system and also restricts the method’s applicability to just the solid/liquid interface. Variation of the backing medium (solvent) is easier to perform, but the inversion technique only works in cases where the film under study is not penetrated by solvent. Additionally, the solubility of the inverse problem requires non-negativity of the SLD profile. All these characteristics impose restrictions for the broad applicability of direct inversion methods.

Several different ‘model-independent’ approaches for the reconstruction of interfacial structure from reflectivity measurements have been reported, based on either numerical or stochastic methods. Pedersen (1992 ▸) developed a two-step method of an indirect Fourier transform (IFT) followed by square-root deconvolution to obtain an SLD profile. Hohage *et al.* (2008 ▸) introduced an iterative algorithm for profile reconstruction based on regularization methods. Kunz *et al.* (1993 ▸) and Laub & Kuhl (2006 ▸) combined aspects of simulated annealing with special parameterization of the SLD profile, de Haan & Drijkoningen (1994 ▸) used genetic algorithms for model fitting, and Sivia *et al.* (1991 ▸) implemented maximum entropy methods for obtaining ‘free-form’ solutions, while Zhou & Chen (1993 ▸) proposed a groove-tracking method for SLD reconstruction. A common feature of all these approaches is that they treat the case of single contrast measurements that cannot yield a unique density profile, while successful reconstructions usually depend on a good initial guess about the profile or on constraints based on prior knowledge about the system.

In the present work, by combining aspects of previous studies we have developed an integrated methodology for the determination of the hydration (solvent volume fraction) and SLD profile at the air/liquid or solid/liquid interface by multiple solvent contrast neutron reflectometry, without the need for any assumptions concerning the form of the interfacial profiles. The method, which is reminiscent of *ab initio* shape recovery from small-angle scattering data (Svergun, 1999 ▸; Koutsioubas & Pérez, 2013 ▸), is based on the IFT approach developed by Pedersen (1992 ▸), which permits an estimation of the maximum extension of an interfacial layer. Using the maximum extension thus found, and also by applying minimal physical boundary conditions to the hydration and SLD profiles, a simulated annealing search for interfacial profiles is performed, which leads to a satisfactory fit of the reflectivity curves. Through extensive testing with simulated and experimental data, we show that three different solvent contrasts may give reliable reconstructions that are informative about the molecular distribution at an interface for many different types of system. Limitations related to finite spatial resolution and to the potential presence of layers exhibiting labile hydrogen exchange are identified and discussed.

## 2. Materials and methods   

### 2.1. Samples and experimental details   

The lipid 1,2-dioleoyl-*sn*-*glycero*-3-phosphocholine (DOPC) was purchased from Avanti Polar Lipids in the form of lyophilized powder and was used without further purification. Lyophilized hen egg lysozyme protein and Ludox HS-40 colloidal silica were purchased from Sigma–Aldrich. Deuterium oxide (D_2_O) of 99.8% purity was purchased from ARMAR (Europa) GmbH. Unilamellar vesicles were prepared by sonication as described previously (Koutsioubas, 2016 ▸), and were later fused onto hydrophilic substrates to obtain supported membranes. Ultra-polished Si blocks (r.m.s. roughness 1–2 Å, dimensions 150 × 50 × 20 mm) purchased from Andrea Holm GmbH were used as substrates and were cleaned before each experiment using a UV–ozone chamber.

Neutron reflectivity data were acquired on the MARIA vertical reflectometer (Mattauch *et al.*, 2018 ▸) operated by Jülich Centre for Neutron Science at Heinz Maier-Leibnitz Zentrum in Garching (Germany), using custom temperature-regulated liquid cells. The measurements were performed using two different wavelengths, 10 Å for the low-*q* region and 5 Å for the high-*q* region up to 0.25 Å^−1^, with a wavelength spread Δλ/λ = 0.1. The change of solvent contrast in the liquid cells was performed using a combination of valves and a peristaltic pump, at small flow rates ≤0.5 ml min^−1^.

### 2.2. Algorithm   

An interfacial layer of thickness *D* between semi-infinite fronting (solid or air with SLD ρ_0_) and backing (liquid with SLD ρ_solvent_) media is modelled as a succession of *N* = 50 equally thick (*d*) layers, so that *D* = *Nd*. These layers are characterized by a non-solvent component SLD ρ_*n*_ and by a hydration (solvent volume fraction) *h*_*n*_, which can assume values between 0 and 1 for no or full hydration, respectively. A boundary condition is set on the hydration values related to the extremes of the interfacial layer, where *h*_0_ = 0 and *h*_*N*+1_ = 1, since hydration should approach zero near the fronting medium and 1 as we approach the backing medium.

The non-solvent component SLD ρ_*n*_ and hydration *h*_*n*_ of each layer *n* are smeared by a Gaussian error function (Danauskas *et al.*, 2008 ▸) so that at a distance *z* from the fronting medium they take the form
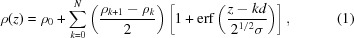


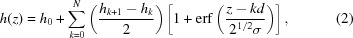
where σ is a smoothing parameter that, as will be seen below, is related to the spatial resolution of the experimental technique. Given equations (1) and (2), the composite SLD ρ′(*z*) is given by the relation
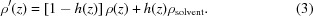
In turn, the theoretical reflectivity is calculated using the Abelès matrix formalism (Abelès, 1950 ▸), where the Fresnel reflection coefficient between layers *n* and *n* + 1 is given by
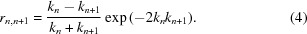

*k_n_* = {*q* ^2^/4 − 4π[ρ′(*nd*) − ρ_0_]}^1/2^ is the neutron wavevector in layer *n*. For each layer a characteristic matrix is defined as
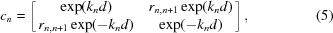
and the system’s matrix *M* is given by
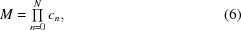
from which finally the reflectivity is calculated as
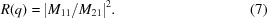



The finite instrumental resolution δ*q*/*q* is taken into account by assuming a Gaussian resolution function that is convoluted with the theoretical curves using the following equation for performing a *p* point average:
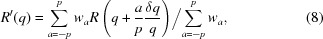
with *w*_*a*_ being the Gaussian weight
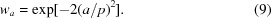



The search for a smoothed non-solvent component SLD and hydration profile that reproduces the experimentally measured reflectivity curves starts from a random ρ_*n*_, *h*_*n*_ distribution. The smoothing parameter σ is initially set equal to π/4*q*_max_. The agreement between the model and the measured data is given by the following score function:
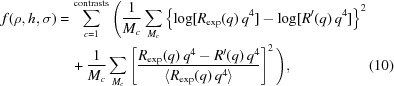
where the angle brackets represent the mean value of the *q* ^4^-multiplied experimental curves and *M*_*c*_ is the number of measurements for each curve with different solvent contrast. The defined score function *f* has essentially two contributions, one from the squared difference between the experimental and theoretical *q* ^4^-weighted reflectivities and a second one from the squared difference of their logarithms. Many different forms of *f* have been tested, including log*R*, *Rq* ^4^, log*Rq* ^4^ and 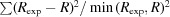 (Kunz *et al.*, 1993 ▸). It was found that while *Rq* ^4^ gave the best results, there is some noticeable bias towards high *q*. It was empirically found that by defining a composite score function as in equation (10) the entire *q* range is handled evenly. We note that, using this form of *f*, negative reflectivity values that may arise after background subtraction cannot be considered. For a related discussion about potential fit bias one may refer to a recent report (Kwaambwa *et al.*, 2010 ▸)

The algorithm proceeds by performing three types of trial modification: (i) a random change of ρ_*n*_ of a random layer *n* within the defined limits ρ_min_, ρ_max_; (ii) a random change of *h*_*n*_ of a random layer *n*; or (iii) a random change of the smoothing parameter within the limits π/4*q*_max_ ≤ σ ≤ π/2*q*_max_ which represent the estimated limits of the experimental resolution as defined by the maximum measured value of the wavevector transfer [see ch. 12 of Fitter *et al.* (2006 ▸)]. Since a simulated annealing scheme is used for the minimization of the score function, each attempted trial is accepted if Δ*f* < 0 or if Δ*f* > 0 with a probability exp(−Δ*f*/*T*), where *T* is the annealing temperature. For the results presented in this work the starting temperature was set to *T* = 1, and 100*N* trials are attempted before reducing the temperature using the schedule *T*′ = 0.9*T*. The annealing stops when 100 attempted changes in a row are rejected.

An important parameter in the described model is the overall extension of the interfacial layer *D*. In order to estimate *D* reliably we use the indirect Fourier transform (IFT) approach introduced by Pedersen (1992 ▸), which gives an estimate of both the ideal (regularized) reflectivity curve and the profile correlation function *p*(*z*) of the derivative dρ/d*z* of the SLD. In more detail, *p*(*z*) is approximated as a series of cubic *b* spline basis functions which are defined in the range 0 ≥ *z* ≥ *D*. The coefficients of the spline basis functions are determined by a constrained weighted least-squares minimization so that the Fourier-transformed and instrumental-resolution-smeared series finally represents the regularized approximation of the experimental data. The term ‘constrained’ refers to the addition of a term *N*_*c*_ multiplied by Λ (Lagrangian multiplier), which can be determined by the point-of-inflection method (Pedersen, 1992 ▸). Λ ties the coefficients of the *b* splines together and leads to a smooth final solution.

In practice, the estimation of *D* when no *a priori* information is available involves trial IFT calculations using arbitrary *D* values until a satisfactory description of the experimental data is obtained and *p*(*z*) approaches the *z* axis smoothly at *z* = *D* (Glatter, 1977 ▸; Müller & Glatter, 1992 ▸). In the next section we will present example applications of IFT calculations using the point-of-inflection method, which lead to reliable estimations of the overall layer extension that is later used as a fixed parameter for the simulated annealing procedure.

## 3. Results   

In this section we present a series of interfacial profile reconstructions from simulated and experimental multi-contrast sets of reflectivity curves at the Si/water interface. These reconstruction serve as a validation of the capabilities of the described methodology. In all cases, ten simulated annealing runs were executed and the model with the best agreement with the experimental data was considered as the final solution. In the generation of simulated reflectivity curves we keep points up to *q* = 0.25 Å^−1^ and down to reflectivity values of 10^−6^, which is the usual background limit for neutron reflectometers, and we also assume a δ*q*/*q* resolution of 10%. The minimum ρ_min_ and maximum ρ_max_ of the profiles are set equal to the values for H_2_O and D_2_O, respectively, since no deuterated molecules were present in our simulated and experimental systems. For most aqueous systems, these H_2_O and D_2_O SLD values will represent the minimum and maximum values that should be used. Only when deuterated substances are present in an experiment should the maximum SLD limit be set accordingly (as an example we can mention deuterated lipids, where the SLD may well exceed 6.35 × 10^−6^ Å^−2^, which is the SLD value for D_2_O). For selected examples we also outline the IFT calculations that lead to the estimation of the profile extension *D*.

### 3.1. Reconstructions from simulated data   

We begin with a hypothetical three-layer system on silicon, where each layer has a thickness of 50 Å and a roughness of 5 Å. As we move away from Si the non-solvent component SLD of each layer is equal to 5 × 10^−6^, 0 × 10^−6^ and 2 × 10^−6^ Å^−2^, respectively. Additionally, we let the third layer be 50% penetrated (hydrated) by the solvent. The three-layer structure is in contact with water and curves for three different contrasts were generated: 100% D_2_O (D_2_O), 38% D_2_O (silicon-matched water, SMW) and 0% D_2_O (H_2_O).

**Figure 1:**
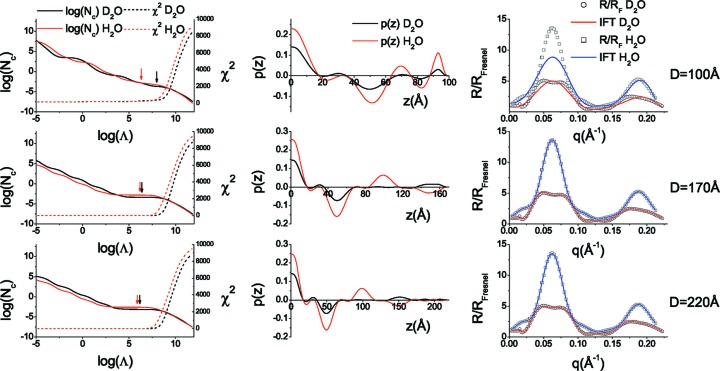
IFT calculations for *D* = 100, 170 and 220 Å for the simulated three-layer system. (First column) Stability plots for the determination of Λ. (Second column) Obtained profile correlation functions *p*(*z*). (Third column) Input data versus calculated regularized reflectivity. Note that the system’s reflectivity is divided by the Fresnel curve corresponding to the Si/water interface.

We applied the IFT methodology for the estimation of the overall layer extension *D* for the D_2_O and H_2_O contrasts. In Fig. 1 ▸ we illustrate the results of IFT calculations for three different trial *D* values. We begin by inspection of the stability plot [log(*N_c_*) and χ^2^ versus log(Λ)] and identify the inflection point of log(*N_c_*) versus log(Λ), which is the middle of the plateau before χ^2^ starts to increase (Pedersen, 1992 ▸; Glatter, 1977 ▸; Müller & Glatter, 1992 ▸). Next, we can identify the effects of under- or overestimating *D* on the calculated profile correlation functions and regularized reflectivity curves. For *D* = 100 Å, *p*(*z*) tends to oscillate and the agreement between the input and calculated regularized reflectivity curves is quite poor. On the other hand for *D* = 220 Å, the input and regularized reflectivity curves are in agreement, although *p*(*z*) fluctuates around the *z* axis for *z* > 170 Å. The middle value, *D* = 170 Å (which is slightly larger than 150 Å owing to the introduced roughness in the input model), provides agreement between the input and calculated regularized reflectivity, while *p*(*z*) approaches the *z* axis smoothly at *z* = 170 Å. This procedure for estimating *D* is similar to that used for small-angle scattering data (Glatter, 1977 ▸) and guidelines for its application have been discussed in detail by Müller & Glatter (1992 ▸).

**Figure 2:**
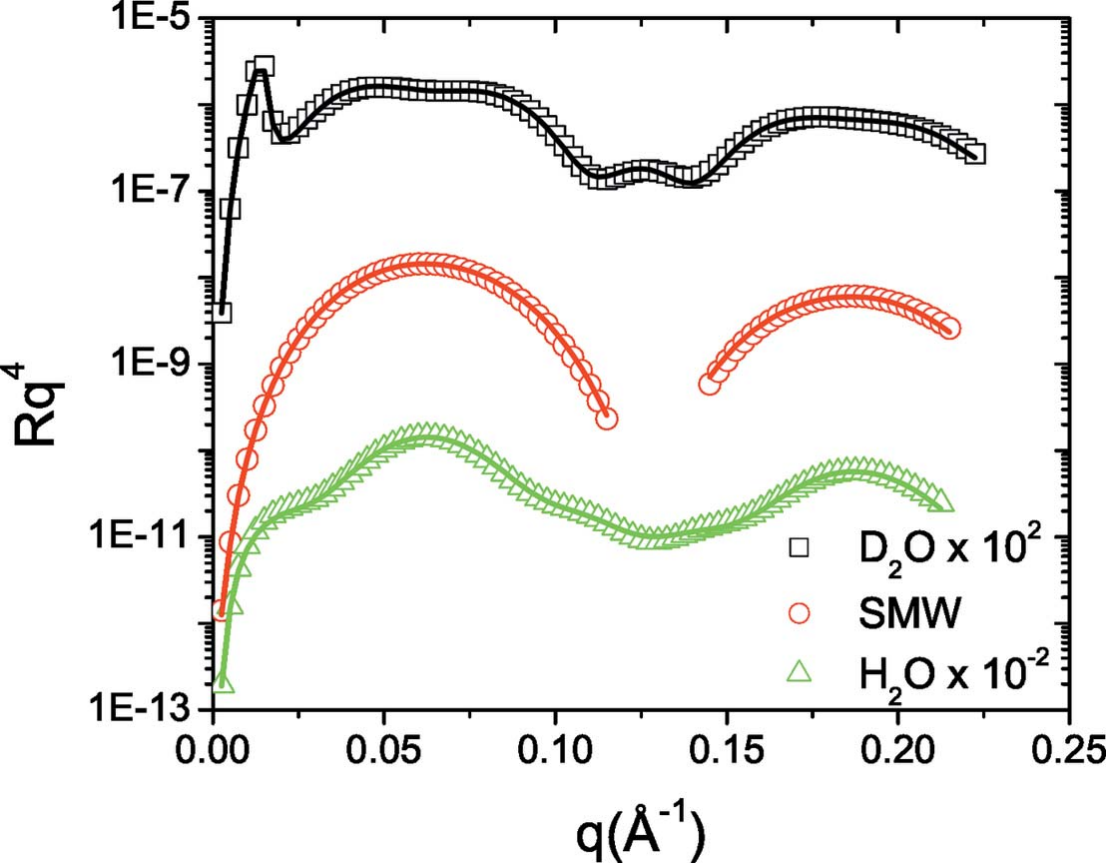
Input reflectivity data for the simulated three-layer system at three different solvent contrasts (points) and the corresponding fitted reflectivity curves (solid lines). For clarity, the D_2_O and H_2_O data are shifted by two orders of magnitude. The gap that appears in the SMW contrast data arises because we do not consider reflectivity values below 10^−6^.

**Figure 3:**
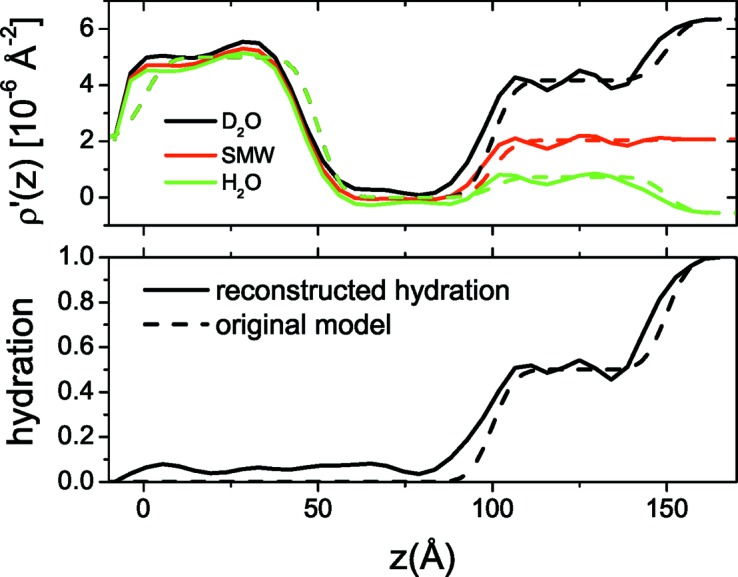
(Top) Composite SLD and (bottom) hydration profiles for the three-layer system. Solid lines represent the reconstructed profiles and dashed lines the profiles of the original model.

By fixing *D* to the value of 170 Å the simulated annealing algorithm is applied to the three sets of input solvent contrast data. In Fig. 2 ▸ we illustrate the fit obtained by the algorithm, and in Fig. 3 ▸ we plot the final calculated ρ′(*z*) and *h*(*z*) profiles compared with the input theoretical ones. The input data are well reproduced by the final fitted curves over the entire *q* range. Additionally, both profiles reflect the structural features of the initial model system. The reconstructed hydration of the first two layers is close to zero (<10%) and the final layer is found to be 50% hydrated, as expected. The overall thickness and composite SLD of each layer are also in agreement with expected values. Small oscillations of the reconstructed profiles around the theoretical ones are related to the limited spatial resolution due to the finite *q* range that is accessible by neutron reflectivity. The final fitted smoothing parameter of the model (σ) was found to be 6 Å.

We now move to another example involving simulated input data, which concerns a thicker parabolic SLD profile at the silicon/water interface that may be produced by a close-packed arrangement of spherical silica nanoparticles (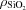 = 3.5 × 10^−6^ Å^−2^) of 250 Å radius at the silicon surface. After the calculation of reflectivity curves for the three different contrasts, IFT calculations suggested *D* = 500 Å for the nanoparticle layer, as expected from the diameter of the particles.

**Figure 4:**
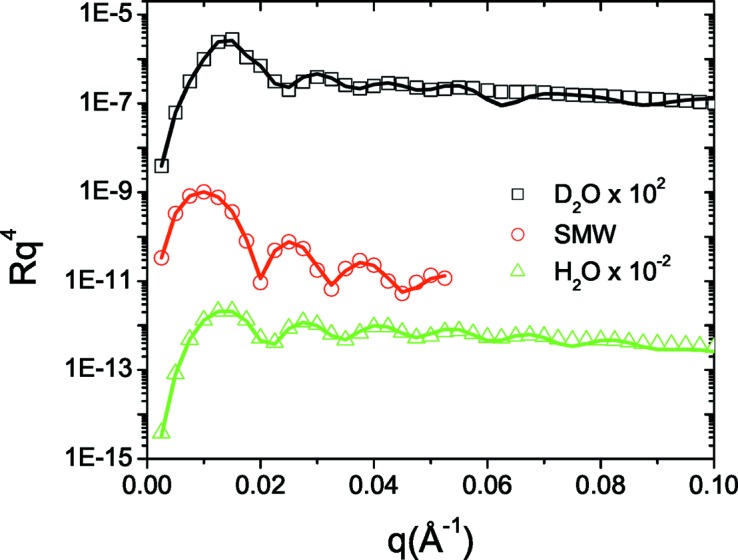
Input reflectivity data for the simulated silica nanoparticle on silicon system at three different solvent contrasts (points) and the corresponding fitted reflectivity curves (solid lines). For clarity, the D_2_O and H_2_O data are shifted by two orders of magnitude.

**Figure 5:**
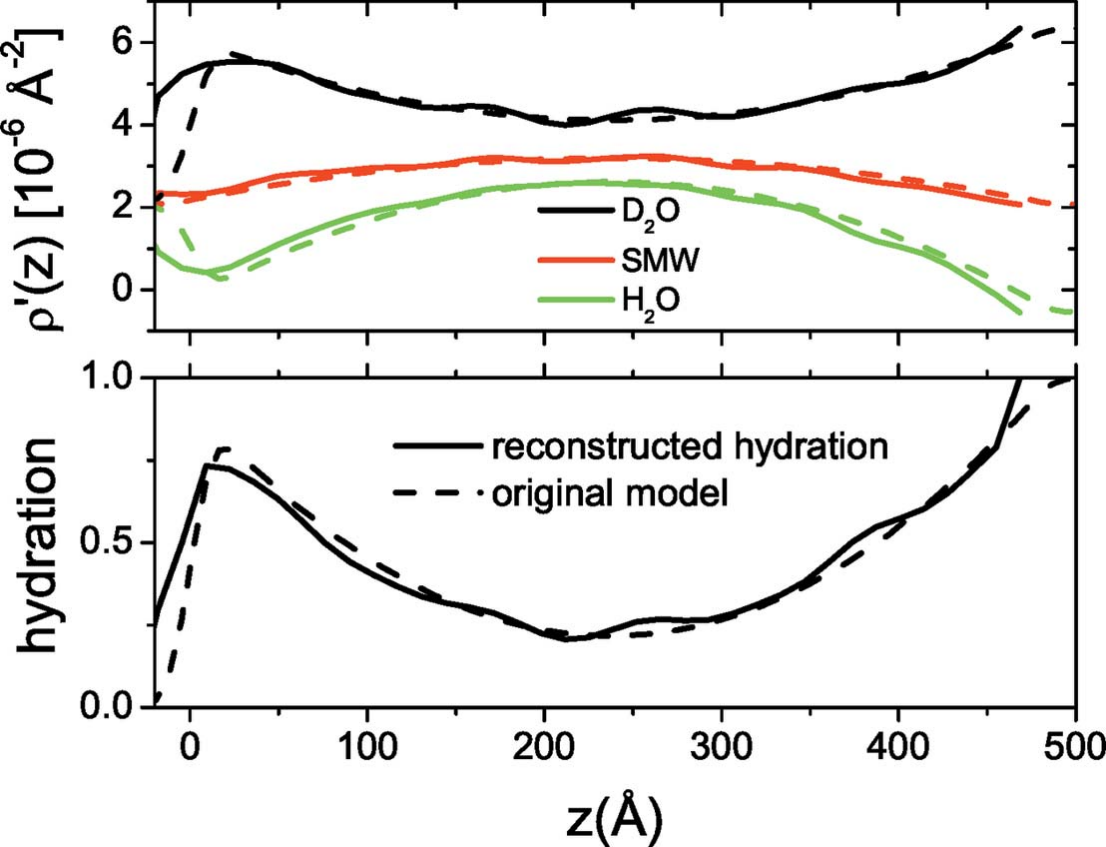
(Top) Composite SLD and (bottom) hydration profiles for the silica nanoparticle on silicon system. Solid lines represent the reconstructed profiles (σ = 15 Å) and dashed lines the profiles of the original model. Calculation of the original model reflectivity curves was performed by approximating the parabolic profile with 20 layers and by assuming a layer roughness of 10 Å under the Névot–Croce approximation (Névot & Croce, 1980 ▸).

Application of the simulated annealing algorithm resulted in a good fit to the data (Fig. 4 ▸), while the reconstructed composite SLD and hydration profiles (Fig. 5 ▸) reliably capture the parabolic structure of the model layer, with only some small deviations close to the region of steep change close to the silicon substrate. Here we note that the success obtained in the ρ′(*z*) and *h*(*z*) reconstructions was also observed in many other case studies (results not shown) involving simulated systems of supported lipid bilayers, polymer brushes on surfaces and lipid monolayers at the air/water interface.

### 3.2. Reconstructions from experimental data   

Having established the robustness of the coupled IFT/simulated annealing methodology in the reconstruction of SLD and hydration profiles from multi-contrast reflectivity data of simulated model systems, we proceed to the application of the method to actual experimental data from the silicon/water interface. In all cases, incoherent scattering and background-corrected experimental data were used. Because we know beforehand that there is a native 10 Å-thick oxide layer on the silicon substrate with low water penetration, we apply two constraints in the first 10 Å above the surface: (i) the hydration should be zero in that region and (ii) the non-solvent component SLD should be equal to 3.5 × 10^−6^ Å^−2^.

The first experimental system concerns the adsorption of a protein (hen egg lysozyme) on silica (the native hydrophilic nanometre-thick layer on silicon). This system has been also studied in the past using multi-contrast neutron reflectometry (Su *et al.*, 1998 ▸) and offers a chance to compare the conclusions of this study with the results from the proposed model-free fitting method. Therefore, during the experimental procedure we maintained similar experimental conditions to those described in the previously published work (Su *et al.*, 1998 ▸), *i.e.* a protein concentration of 1 mg ml^−1^ in 0.02 *M* NaCl at pH 7.

**Figure 6:**
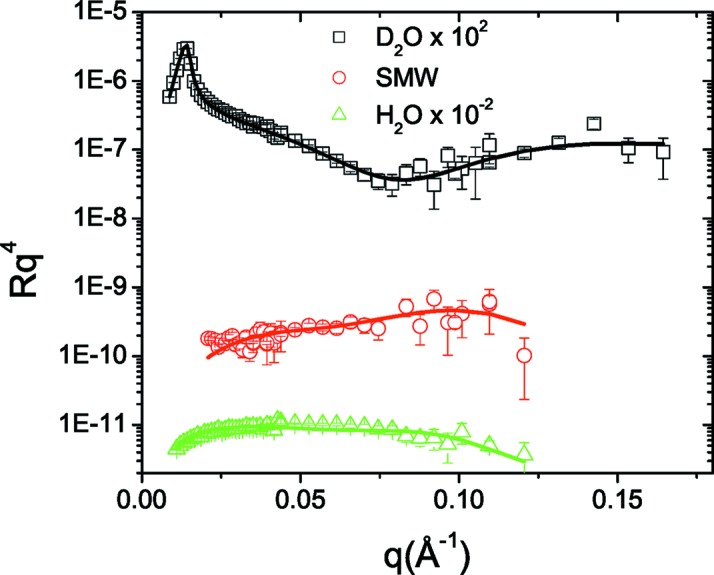
Experimental reflectivity data for lysozyme adsorption on silica at three different solvent contrasts (points) and the corresponding fitted reflectivity curves (solid lines). For clarity, the D_2_O and H_2_O data are shifted by two orders of magnitude.

**Figure 7:**
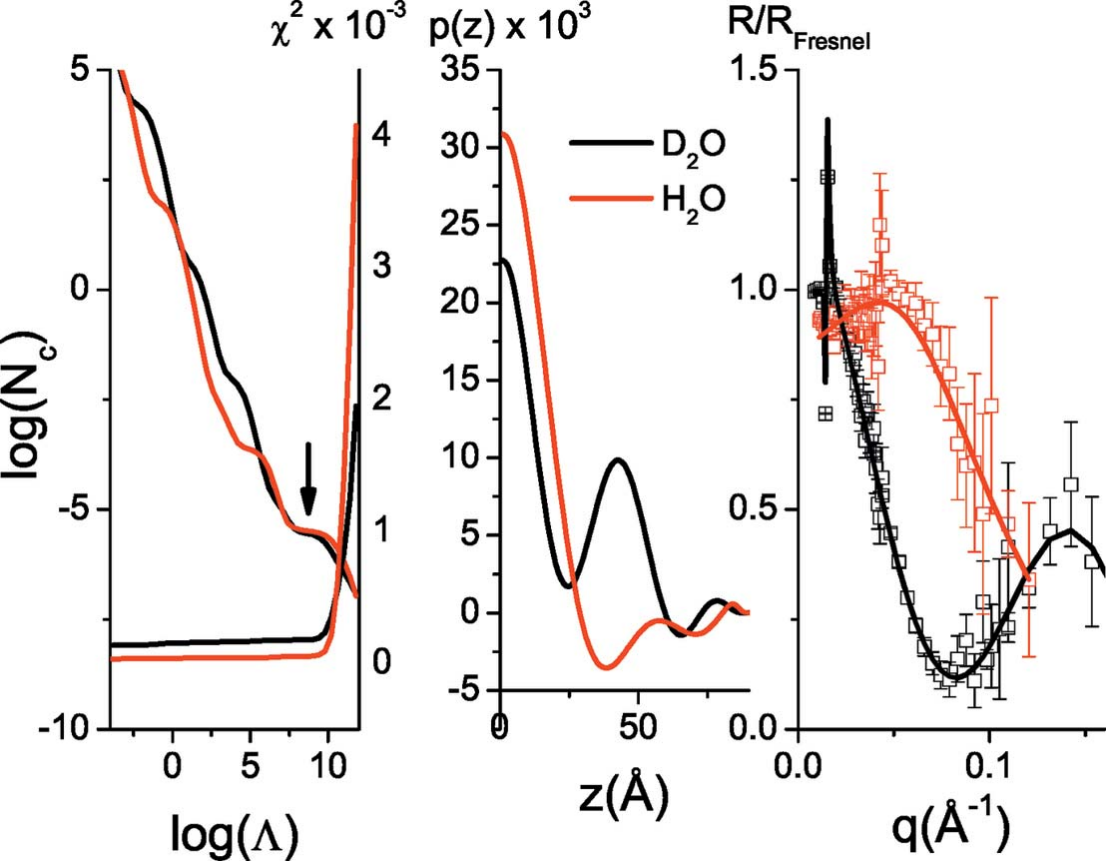
IFT calculations for *D* = 90 Å for the adsorbed protein layer system. (Left) Stability plot for the determination of Λ. The arrow points to the found optimum Λ value. (Centre) Obtained profile correlation functions *p*(*z*). (Right) Input data versus calculated regularized reflectivity. Note that the system’s reflectivity is divided by the Fresnel curve corresponding to the Si/water interface.

**Figure 8:**
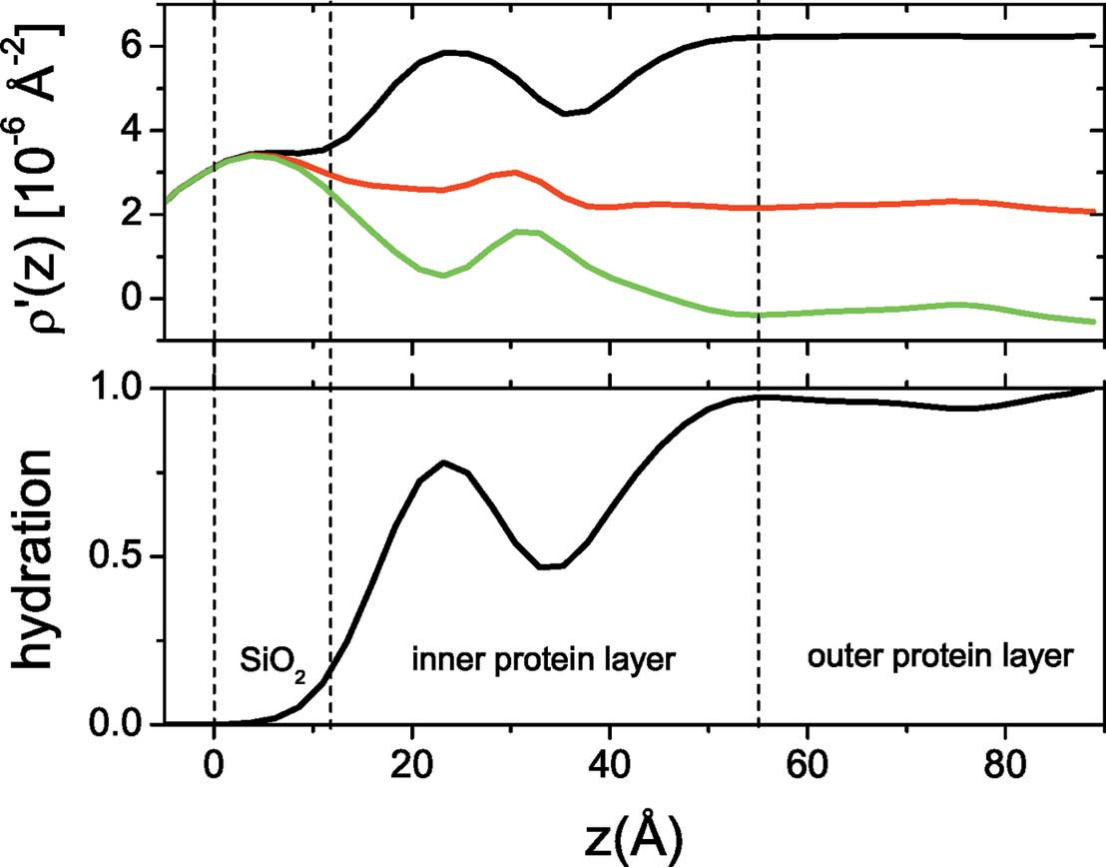
Composite SLD (top) and hydration (bottom) profiles of the adsorbed lysozyme layer (σ = 4.8 Å). Dashed lines indicate the layering of the system: silicon/silica/dense protein layer/dilute protein layer.

In Fig. 6 ▸ the experimental data for lysozyme adsorption on silica are plotted. By applying the IFT on the D_2_O and H_2_O curves (Fig. 7 ▸) we estimate that *D* is 90 Å, since for this value we get a good agreement between the experimental and regularized reflectivity and *p*(*z*) also approaches the *z* axis smoothly at *z* = *D*. We then reconstructed the ρ′(*z*) and *h*(*z*) profiles of the system (Fig. 8 ▸), a procedure that also gives satisfactory fits to the experimental data (Fig. 6 ▸). Inspection of the obtained profiles reveals the following general features. The adsorbed proteins form two layers, one dense and close to the surface, ∼40 Å thick and with a protein volume fraction of about 40%, and a second much more dilute layer, ∼30 Å thick and with a protein volume fraction of about 10%.

It is quite interesting that the study by Su *et al.* (1998 ▸) under the same experimental conditions suggests a similar molecular distribution at the interface, *i.e.* two protein layers with thicknesses identical to the ones found here. Given the molecular dimensions of the lysozyme protein (30 × 30 × 45 Å), the protein molecules in the first dense layer assume a conformation with their long axis inclined to the surface, while in the second layer the protein long axis seems to be parallel to the surface. However, the protein volume fractions in both layers appear to be smaller in the present fits by 10–15%. We attribute this discrepancy to the fact that in our model the exchange of protein labile hydrogen upon contrast change is not taken into account,^1^ thus leading to a slight overestimation of protein layer hydration, or equivalently to an underestimation of protein volume fractions. In principle, this effect could have been taken into account by defining a ρ_solvent_-dependent non-solvent component SLD ρ_*n*_, but this additional free parameter and the fact that the exchange of labile hydrogen atoms and the change in a layer’s hydration tend to influence the composite SLD in the same way would have made the model more complicated, while at the same time affecting solution stability and convergence in the general case. Nevertheless, this is a limitation under the current formulation of the method that should be kept in mind when performing experiments with molecular species that potentially contain labile hydrogen.

**Figure 9:**
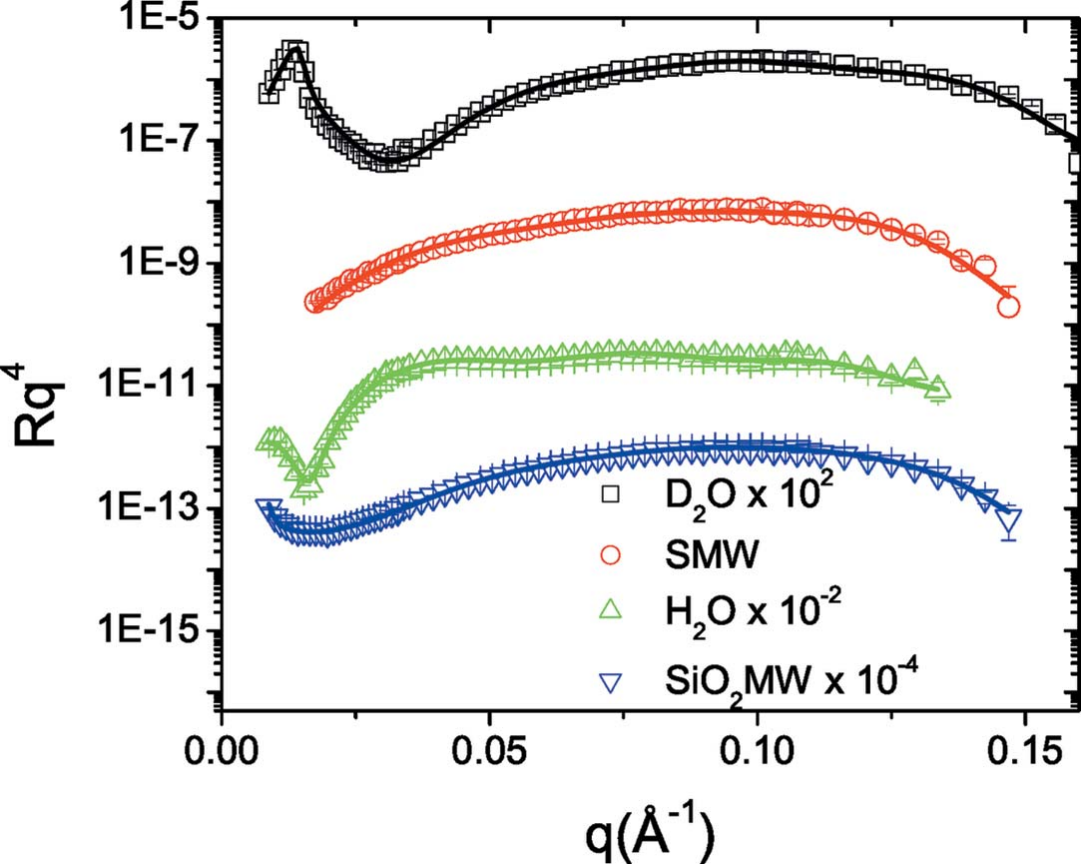
Experimental reflectivity data for the DOPC/silica nanoparticle system at four different solvent contrasts (points) and the corresponding fitted reflectivity curves (solid lines). For clarity, the D_2_O, H_2_O and SiO_2_MW data are shifted as indicated in the legend.

The next example with actual experimental data involves a more elaborate system of silica nanoparticles (Ludox HS-40, diameter ≃ 120 Å) adsorbed on top of a DOPC supported lipid membrane on the native silica layer of a silicon substrate. The realization of the system included the following steps: (i) the fusion of DOPC vesicles on silica; (ii) rinsing of the measurement cell; (iii) injection of the silica nanoparticle solution; and finally (iv) another rinse with solvent. Owing to the hydrophilic nature of both phospholipid heads and silica, the nanoparticles are expected to have a high affinity for the membrane surface. Neutron reflectivity measurements at four different solvent contrasts were performed (Fig. 9 ▸), namely D_2_O, SMW and H_2_O as for the previous example, and additionally SiO_2_MW (silica-matched water) with 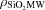 = 3.5 Å^−2^. IFT calculations gave an overall layer extension of *D* = 220 Å.

**Figure 10:**
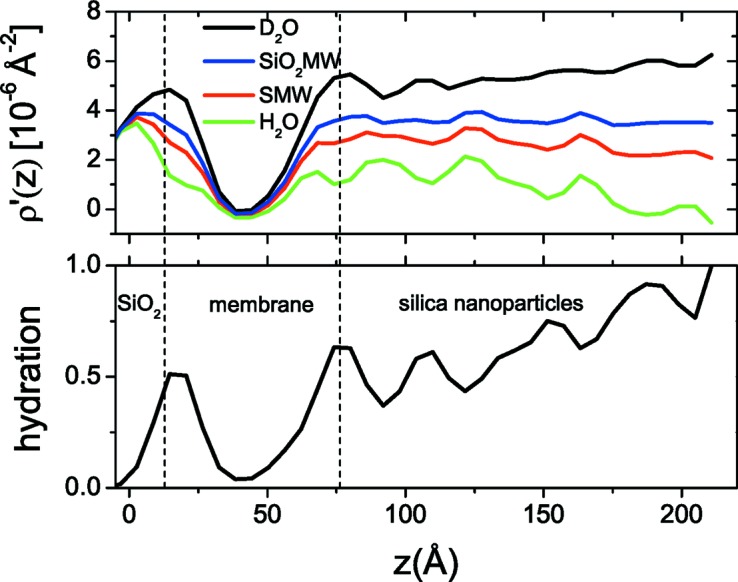
(Top) Composite SLD and (bottom) hydration profiles of the DOPC/silica nanoparticle system (σ = 6.2 Å). Dashed lines indicate the layering of the system: silica/DOPC membrane/silica nanoparticles.

The simulated annealing reconstruction using all four contrasts resulted in the hydration and SLD profiles presented in Fig. 10 ▸, where we may easily distinguish two main structural features after the native SiO_2_ layer: (i) a low hydration region close to the substrate with marginally negative composite SLD values in its centre and (ii) a thick layer (∼140 Å) on top with decreasing hydration as we move towards the bulk solvent. The first layer represents the DOPC membrane and has a thickness of ∼60 Å, which is compatible with the sum of the thickness of a hydration layer between silica and the lower membrane leaflet (∼10 Å) plus the thickness of a DOPC membrane (∼50 Å). The negative ρ′(*z*) values in the centre of the layer and the low hydration are indicative of the presence of a dense hydrophobic hydrocarbon core or lipid tails. The second layer has a relatively constant non-solvent component SLD equal to 3.7 ± 0.35 × 10^−6^ Å^−2^ (quite close to the expected value for silica) and a slightly larger thickness than the diameter of the Ludox nanoparticle (as estimated by dynamic light scattering), most probably due to the polydispersity of the nanoparticles. The overall volume fraction of the layer is 35%, which is quite close to the asymptotic density of the random sequential adsorption model (Meakin & Jullien, 1992 ▸), thus suggesting that Ludox particles, as they approach the upper membrane leaflet and if they do not overlap with other particles, adsorb on the membrane surface and stay fixed at this position.

All reconstructions presented in this section involve at least three solvent contrasts. Tests have also been performed by providing as input only one or two contrasts for each system. The general conclusion is that, except for special cases of ‘simple’ high-contrast profiles, two solvent contrasts are not enough to guide the simulated annealing algorithm to the correct solution. This means that, in the general case, three contrasts represent the minimum number that may provide a reliable interfacial profile reconstruction.

## 4. Discussion   

By reviewing the results of the previous section we may claim that the combination of IFT calculations for the estimation of maximum layer extension, together with the simulated annealing reconstruction of SLD and hydration profiles from at least three solvent-contrast neutron reflectivity measurements, permits the reliable recovery of the interfacial mol­ecular distribution for a series of different systems within the limits of finite spatial resolution and without any *a priori* assumptions. To some extent, the proposed method shares common concepts with *ab initio* shape-recovery methods from small-angle scattering data (Svergun, 1999 ▸; Koutsioubas & Pérez, 2013 ▸), with the difference here being that a 1D SLD and hydration profile is reconstructed.

An important aspect of the IFT/simulated annealing approach compared with model fitting is that no bias towards a specific form of final solution is imposed. In model-fitting algorithms a search of parameter space is performed within the limits of a layer model suggested by theory or intuition, while here the SLD and hydration profiles are left free to explore any form between the substrate and the maximum layer extension found by IFT. In this respect it can be stated that the maximum amount of objective information concerning the interface is recovered.

The correct estimation of the maximum extension *D* is a crucial step that limits the computational effort needed and also aids the convergence of the method to a correct and stable solution. In cases where a reliable estimation of *D* is available from an independent experimental technique, or from previous knowledge about the system or by using alternative estimation methods like the ‘Guinier’ approximation (Dickinson *et al.*, 1993 ▸; Henderson *et al.*, 1993 ▸), the IFT step can be bypassed and one can proceed directly with the simulated annealing reconstruction of the interfacial profile.

In contrast to previous attempts to solve the reflectivity inverse problem using stochastic methods (Laub & Kuhl, 2006 ▸; Kunz *et al.*, 1993 ▸; Zhou & Chen, 1993 ▸; Sivia *et al.*, 1991 ▸), here we take advantage of the concurrent fit of multiple solvent contrasts, thus avoiding the need for a ‘good initial guess’ for the profiles or for a set of criteria for identifying physically reasonable profiles among different candidates. Despite the fact that only examples at the solid/liquid interface were showcased in the previous sections, the methodology is also directly applicable at the air/liquid interface without any modifications. Additionally, there is no need for special reference layers (Majkrzak *et al.*, 2000 ▸), thus avoiding the need for special experimental setups and extensive pre-characterization of the system. In this sense, the method has general applicability since multiple solvent-contrast neutron reflection experiments are performed routinely with neutron reflectometers around the world.

On the basis of these facts, we expect that the presented algorithm may assume a complementary role to traditional model fitting, and more specifically might offer solutions in the following general cases:

(i) It could provide reconstructed profiles when an interface presents complicated behaviour that is difficult to express in the form of several layers whose SLD and hydration values can be represented by an analytical function.

(ii) When absolutely no information about the interface is known, it could be used to provide hints for building a layer model that can be refined later through model fitting.

(iii) During a neutron reflectivity experiment, when decisions have to be taken about the quality of a sample and the continuation of the experiment, the ability to obtain a fit with minimal human intervention might prove beneficial.

### 4.1. Effects of limited resolution   

In some of the presented profile reconstructions, and especially in regions where we expect plateaux (Fig. 3 ▸) or a smooth variation of the profile (silica nanoparticle layer on DOPC, Fig. 10 ▸), we observed some ‘ripples’ that might seem artificial. In the case of simulated examples, we have generated reflectivity curves at *q*_max_ values that are much higher than what is experimentally accessible in neutron reflectivity experiments, in order to identify its effect on the reconstructed profiles. It was found that as *q*_max_ increases these ripples disappear and the SLD profiles become smoother and more representative of the original profile. This behaviour indicates that the observed effect is related to the limited spatial resolution due to the finite *q*_max_ in the input curves (data truncation effect; Majkrzak *et al.*, 2000 ▸).

**Figure 11:**
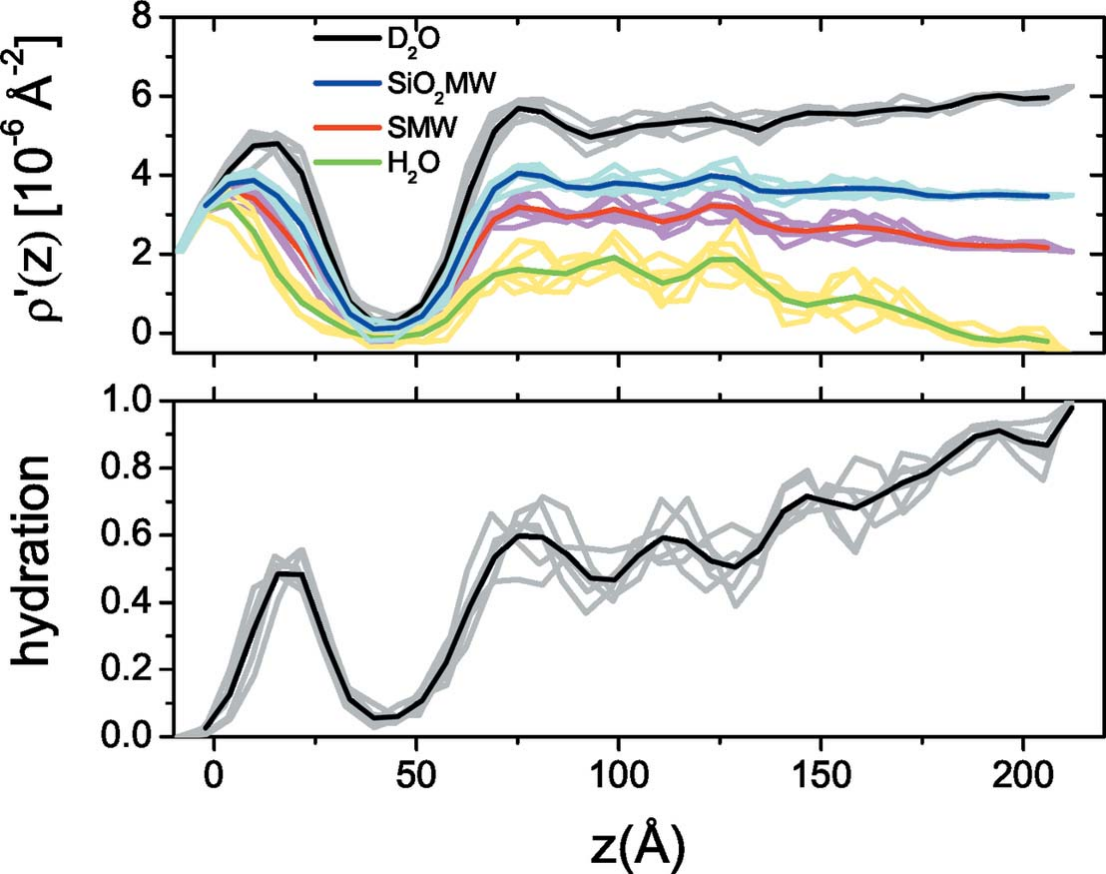
(Top) Composite SLD and (bottom) hydration profiles of the DOPC/silica nanoparticle system. Light-coloured lines represent the profiles obtained by different simulated annealing runs. Solid lines represent the average of multiple runs.

A way of partially suppressing this effect is by taking advantage of the stochastic nature of simulated annealing. In practice, when executing multiple annealing runs, apart from a subset of runs that are trapped in local minima and give visibly inadequate fits of the input data, we obtain a number of solutions with low score function (*f*) values belonging to very similar SLD and hydration profiles that describe the experimental data equally well. By averaging these profiles we get a reduction of the rippling and also an estimate of the variance of the reconstructed profiles. In Fig. 11 ▸ we present the results of the application of profile averaging for six different low-*f* runs of the DOPC/nanoparticle system.

### 4.2. Application of constraints   

In relation to the always present native oxide layer on silicon, we have already encountered the concept of constraint application on the SLD and/or hydration profiles during simulated annealing. From the technical point of view, the application of such constraints involves simply the prohibition of changing specific parts of the SLD or hydration profile during the annealing. When *a priori* information is known concerning the system under study, it can be implemented in the form of constraints that guide the annealing towards more precise reconstructions.

**Figure 12:**
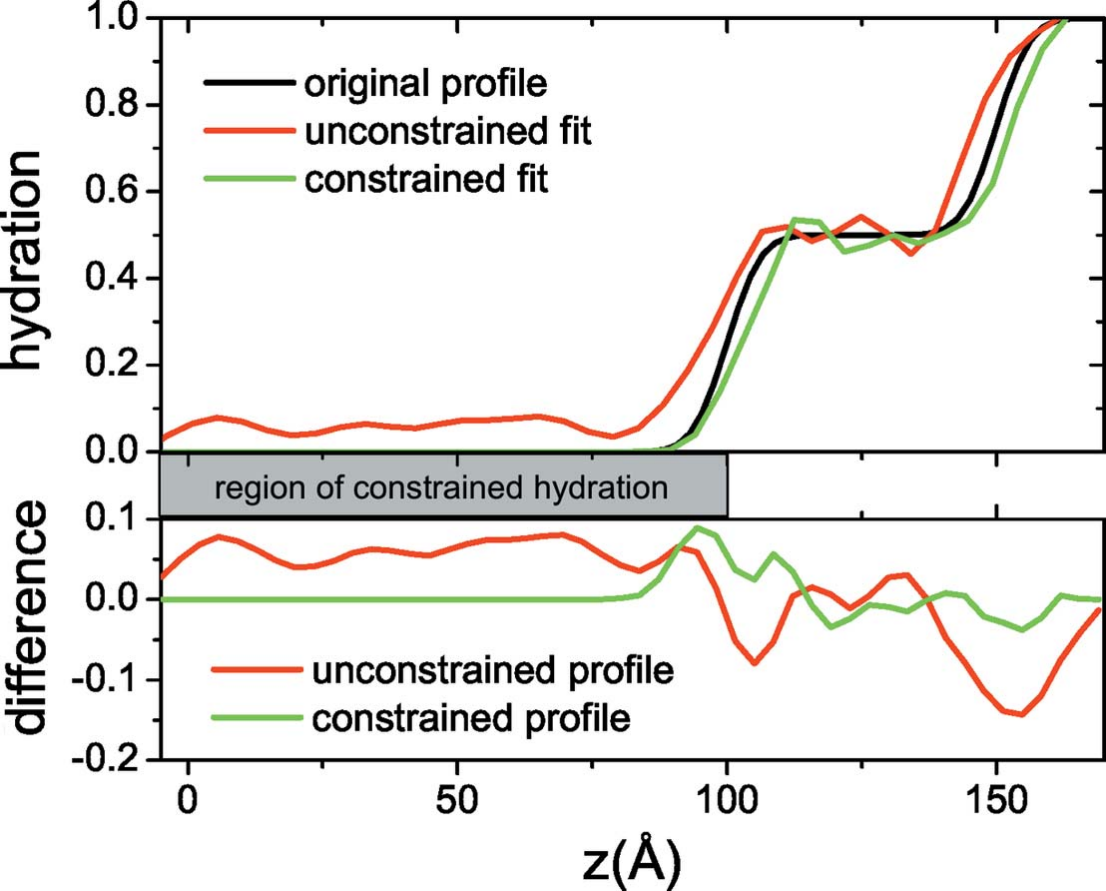
(Top) Reconstructed hydration profiles from unconstrained and constrained simulated annealing runs compared with the original profile. (Bottom) The difference between the two hydration profile reconstructions and the original profile.

For example, let us revisit the simulated three-layer system (Fig. 3 ▸) and let us suppose that we know that the region 0 ≤ *z* ≤ 100 Å is non-hydrated. The constrained annealing results in marginally better fits of the input reflectivity curves, while the hydration (Fig. 12 ▸) is closer to the original profile. Additionally, we have found through testing with multiple sets of simulated data that constraint application in some cases leads to reliable reconstructions, even when less than three contrasts are given as input. However, this largely depends on the type of constraint and on the type of system studied.

The application of more elaborate constraints may even aid in cases where layers with labile hydrogen exchange are present in the system, as we encountered above for adsorbed protein layers on silicon. By applying different non-solvent component protein SLD constraints for each solvent contrast, one may take into account the variation of hydrogen content in the protein and, in principle, avoid overestimation of the protein layer hydration. In future updates of the algorithm the possibility for the application of such constraints will be considered.

### 4.3. Computer program   

The described coupled IFT/simulated annealing methodology has been implemented in the program *DIONYSIA*, written in Fortran 90. Typical single runs with three contrasts as input require between 10 and 30 min on a single core of a modern processor. The executable code of the program (for macOS and Linux platforms) is available as supporting information or from the author on request.

## 5. Conclusions   

In summary, a new method based on IFT calculations and simulated annealing has been developed where, by exploiting the information content of neutron reflectivity data at different solvent contrasts, it is made possible to recover interfacial structure for a series of different systems. The IFT calculations permitted the estimation of the overall layer thickness that is used to bound the search of hydration and SLD profiles via simulated annealing. Minimization of the discrepancy between the calculated and measured reflectivity at three or more solvent contrasts, together with a minimum set of physically meaningful constraints, give reliable profile reconstructions. Given that no assumptions need to be made concerning the system under study, the presented model-free approach may assume a complementary role to the more traditional model-based fitting. A related computer program (*DIONYSIA*) has been developed for performing the described calculations and is made available to the scientific community.

## Supplementary Material

Source code and sample data. DOI: 10.1107/S1600576719003534/kc5089sup1.zip

## Footnotes

1 Using the program *SASSIE* (Sarachan *et al.*, 2013 ▸), we estimate the SLD of lysozyme protein to be 3.25 × 10^−6^ and 1.90 × 10^−6^ Å^−2^ for the D_2_O and H_2_O contrasts, respectively.
