# Supplementary material for: Model-independent recovery of interfacial structure from multi-contrast neutron reflectivity data
Source: J Appl Crystallogr. 2019 Apr 30;52(Pt 3):538–47. doi: 10.1107/S1600576719003534 (PMC6557181; doi:10.1107/S1600576719003534)
Supplement: Supplementary file 1 [file j-52-00538-sup1.zip › manual.pdf]

Brief tutorial on the use of the program `dionysia` for interfacial structure recovery from multicontrast neutron reflectivity data.

March 26, 2019

1 Scope

The program can be used for model-free fitting of multiple solvent contrast neutron reflectivity data from experiments performed at the solid/liquid or air/liquid interface. Data input includes, the actual normalised and background subtracted reflectivity curves, and also the scattering length densities (sld) of the substrate and the solvent. No assumptions concerning the form of the interfacial scattering length density profile or its maximum extension (D) need to be made. An Indirect Fourier Transform (IFT) method is used for the estimation of D, that is later used for bounding the search that is performed by a simulated annealing algorithm for finding an sld and hydration (solvent volume fraction) profile that agrees with the experimental data. For a complete description of the used methodology please refer to the article *Koutsioubas A. (2019) Journal of Applied Crystallography.*

2 Program installation and execution

2.1 Dependencies

Note that `gnuplot` should be installed on the system before running the program. On macOS you can either install a Binary version or use macports or Hombrew to install the port.

2.2 Execution in dialog mode

You can run the program in dialog mode just by giving the command (`./dionysia_mac` on macOS or `./dionysia_linux` on Linux).

2.3 Execution in argument mode

You may run the program in command line argument mode where all the input information can be inserted in an ASCII file. In the distribution of the program you may find a parameter file `param.txt` together with three measurement data files `DOPC_CHOL20_D2O.dat`, `DOPC_CHOL20_SMW.dat`, `DOPC_CHOL20_H2O.dat` that concern actual neutron reflectivity measurements of a DOPC+20% cholesterol membrane on a silicon substrate. To run the example give the command `./dionysia_mac param.txt` for macOS or `./dionysia_linux param.txt` for Linux. You may inspect the file `param.txt` and identify the format of parameter input. Note that the sequence of parameter input is the same as in dialog mode. Also note that IFT calculations can be performed only in dialog mode.

3 Description of program's input and output

In the following we give a brief explanation of **input parameters** in dialog mode and also describe the output generated by the program.

* After launching the program the first requested input concerns the **project name**. All output files will begin with the provided **project name**. Previous output files with the same **project name** will be overwritten.

* **Instrumental resolution** (dq/q): For example, for 10% resolution enter the value 0.1

* **Substrate sld** (10^{-6} Å^{-2}) [default=2.07]: Provide the sld of the fronting medium in 10^{-6} Å^{-2} units. Default value is 2.07 for Silicon, if you give no value as input, 2.07 will be used. If for example you work at the air/water interface provide the value 0.

* **Number of solvent contrasts**: essentially the number of different neutron reflectivity curves that will be used as input. Maximum is equal to 10. For each contrast you will be asked to enter the following data:

  - **Reflectivity data file name**: file name together with file extension.This should be an ASCII file with 3 column format containing q,R,δR data. q should be given in Å^{-1}. Reflectivity data provided to the program should be normalised and background corrected.
  - Provide the **solvent sld** in 10^{-6} Å^{-2} units
  - The program asks if the **layer extension** (D) is known. If that's the case you are asked for the value in Å units. If it is not known then the IFT calculation [1] dialog is initiated (see below).

[1]Note that IFT calculations when the sld of backing and fronting medium is the same, cannot be performed.

$>$ The program asks for an **estimation of layer extension** in Å. Then based on this value the stability plot is calculated. After the calculation is done, the program opens a `gnuplot` window with the stability plot. An arrow in the stability plot indicates the found optimum Λ value. You may close then the window. Note that when the estimation of D is much different than the actual value, the automatic estimation of Λ in the stability plot might be erroneous, and the user should provide the value manually.

$>$ If the suggested Λ **value** is satisfactory then just press enter. If not then give a value based on the plot. Note that the `gnuplot` window contents with the stability plot are also written on disk in the form of a `pdf` and `dat` file ending by `_nc.pdf` or `_nc.dat`.

$>$ Given the Λ value the program opens two `gnuplot` windows with the $p(z)$ and the comparison of experimental and regularised curves. You may close the `gnuplot` windows. Their contents are again written on disk in the form of `.pdf` and `.dat` file ending with `_p(r)` and `_IFT_fit` respectively.

$>$ Now the program asks if the experimental data fit is satisfactory and if the profile correlation function is approaching smoothly the z-axis near the maximum layer extension. If that is the case then you have found D. Otherwise you should suggest a new D value and redo the IFT calculations.

$*$ **Maximum sld** (10^{-6} Å^{-2}) [default=6.35]: When working with water this will always be the one of heavy water except for the case when deuterated molecules are used in the experiment, like for example deuterated lipids. In that case max sld should be set equal to the sld of the molecular species with the highest sld in the system. If nothing is given as input the program uses the default value.

$*$ **Minimum sld** (10^{-6} Å^{-2}) [default=-0.56]: When working with water as the solvent, the corresponding value for H2O should be used (default value).

$*$ **Max smoothing parameter** σ_{max} (Å): this parameter represents the max limit of the smoothing parameter that is related to the spatial resolution based on the measured q_{max}. The default proposed value would work for the majority of cases.

$*$ **Min smoothing parameter** σ_{min} (Å): this parameter represents the min limit of the smoothing parameter that is related to the spatial resolution based on the measured q_{max}. The default proposed value would work for the majority of cases.

$*$ **Slab size** (Å): The size of the slabs used for the "binning" of the sld profile. Using the default value is advised. Note that program execution time increases when smaller slab size values are used.

- **Number of applied constraints** (by default zero). The types of constraints that can be implemented are two. Either hydration or sld constraints. If a non zero value is inserted then the constraint dialog will begin (see below).

  - The program asks for the **start of the constraint** (in Å) in relation to $z = 0$ at the end of the fronting medium.
  - The program asks for the **end of the constraint** (in Å) in relation to $z = 0$ at the end of the fronting medium.
  - The program asks for the **type of the constraint**. 0 for hydration and 1 for sld constraint.
  - The program asks for the **value of the constraint**, that can take values from 0 to 1 for hydration and **sld min** to **sld max** for the sld.

- **Initial annealing temperature** [default=1.00]: Default value of annealing temperature will work for most cases. Only if the annealing starts with a trial/success ratio below 80% then consider to ncrease this value.

- **Annealing schedule** [default=0.90]: Default value will work for most cases. If you encounter problems with the convergence of the annealing to good fits then consider increasing this value to 0.95 or even to 0.99 in challenging reconstructions. However execution time will increase.

- **Trials per slab** at each annealing step [default=100]. Again consider increasing this value only if problems of convergence arise.

- **Number of runs**: Single (0) or multiple runs (1) [default multiple]. By default 10 annealing runs are executed and the best model is reported at the end. However a single run can also be executed.

During the annealing procedure the program reports the annealing temperature (T), the score function value (ScF), the smoothing parameter (SmP) and the success over number of trial ratio. Also at the end of the annealing the χ against experimental errors and the elapsed time are reported. All output is also written on disk in the form of a log ASCII file. During the runs and at each change of annealing temperature a series of files is written on disk so that the user may visualise the evolution of the reconstruction.

- `(project name)_hydration.pdf` and `(project name)_hydration.dat`

  containing the hydration profile

- `(project name)_sld_profile.pdf`
  `(project name)_sld1.dat , (project name)_sld2.dat` ...

  containing the composite sld profiles

– `(project name)_no_hydration_sld_profile.pdf` and
`(project name)_no_hydration_sld_profile.dat`

  containing the sld profile excluding the hydration contribution (non-solvent component sld)

– `(project name)_fit.pdf`
`(project name)_fit1.dat , (project name)_fit2.dat` ...

  containing the fits of the curves in R vs Q representation

– `(project name)_fit_q4.pdf`
`(project name)_q4fit1.dat, (project name)_q4fit2.dat` ...

  containing the fits of the curves in Rq^4 vs q representation

4 A step by step example

Let's assume that we have a set of 2-solvent contrast (in D_2O and H_2O) neutron reflectivity measurements of a lipid bilayer membrane on a silicon substrate. In the following we describe in detail the use of the program `dionysia` for model-free fitting the above described data set. We assume that two files containing the experimental data (in three column format, q,R,δR) are placed in a folder together with the executable of the program.

* We begin by running the program in dialog mode

  `./dionysia_mac`

* after the initial displayed info, the program asks for the project name

```
 Project name........................................  membrane
```

  we give the name (membrane), so that all output file written on disk will begin with this name.

* Then we are asked about the instrument resolution. In this particular case $dq/q = 10\%$.

```
Instrumental resolution (dQ/q).......................  0.1
```

* Then we have to enter the sld of the substrate. In our case since we have used silicon, we just use the default value by pressing `return`.

```
Substrate sld (10^-6/Angstrom^2) [default=2.07].......
```

* Then the input of data for each solvent contrast starts. We choose to begin with the data in D_2O.

```
Input for solvent contrast number:  1

Reflectivity data file name..........................  D2O.dat
Solvent sld (10^-6/Angstrom^2).......................  6.35
```

* After giving the filename of the data file and the sld of the solvent, we are asked if the maximum layer extension (D) is known. If it is known, we just have to enter the value. If not (as we assume in this example), then we are asked for an initial guess and based on this value the IFT procedure starts. Let's start with an initial guess $D = 30$ Å.

```
Is maximum layer extension known?  [1 (yes),0 (no)].....0
Layer extension (Angstrom)...........................  30
Inverse Fourier tranform of experimental curve
Calculating....
```

after the calculation is done, the stability plot pops-up on the screen. In the stability plot we need to identify a $log(\Lambda)$ where there is an inflexion point in the N_c curve, while χ is still small. In this particular stability plot, the automatic procedure fails to identify such an inflexion point (arrow position). Visually we may identify that this inflexion point is positioned at $log(\Lambda) = 7$.

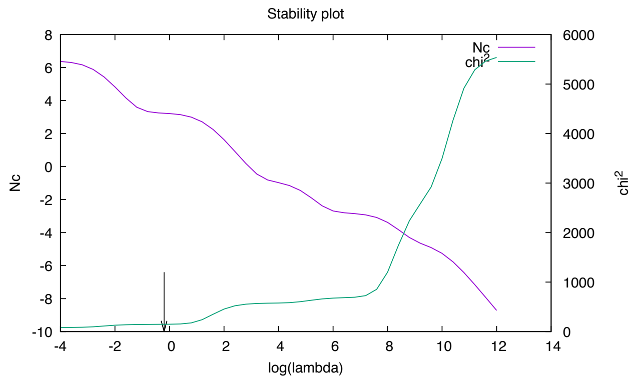

* We then enter $log(\Lambda) = 7$

```
Inspect stability plot and suggest log(lambda) value..  7
```

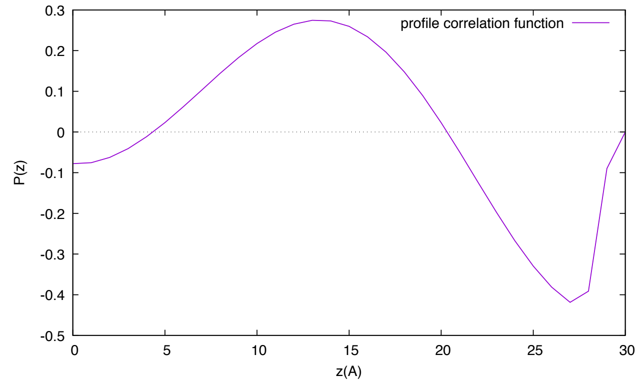

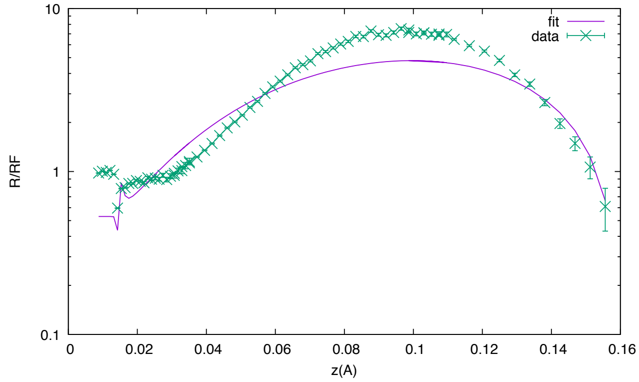

* Using this value the program calculates and displays (pop up windows) the profile correlation function $p(z)$ and the fit of the data.

  We can make two clear observations. a) the data fit is inadequate and b) the $p(z)$ is not approaching the z-axis smoothly at $D = 30\,\text{Å}$. For these reasons we reply negatively in the question of the program

```
Is the experimental data fit satisfactory and the profile
correlation function approaching smoothly the z-axis near
the maximum layer extension?  [1 (yes),0 (no)]...........  0
```

* Since D is not found yet, the program asks again for a guess. Let's put a much larger value that previously, i.e. $D = 100\,\text{Å}$.

```
Layer extension (Angstrom)...........................  100
Inverse Fourier tranform of experimental curve
Calculating.....
```

* This time the automatic procedure finds the inflection point correctly (see stability plot below) and we choose to use the default value pressing return

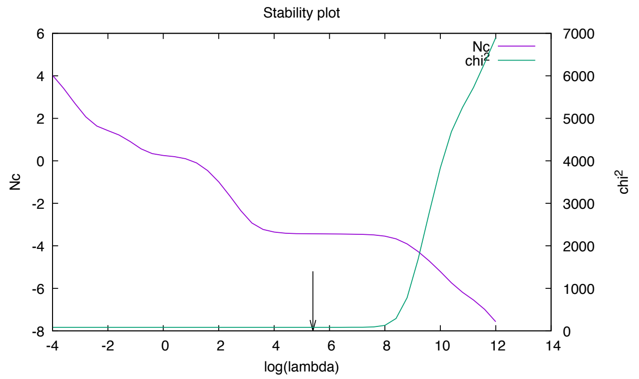


```
Found log(lambda) = 5.4
Inspect stability plot and suggest log(lambda) value..
```

* Then the profile correlation function $p(z)$ and the fit of the data are displayed

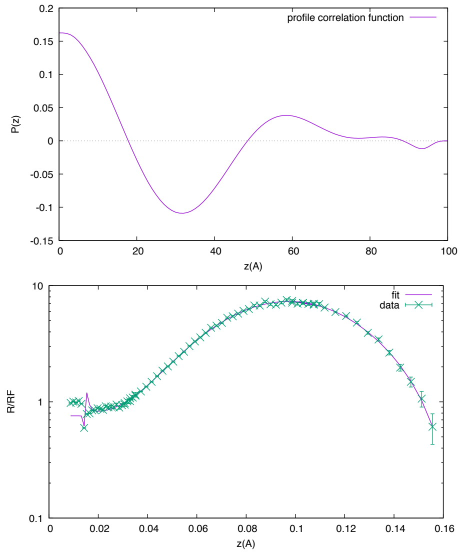

Note that the data fit is now satisfactory. However we note that after around $z = 75\,\text{Å}$, $p(z)$ tends to fluctuate around the z-axis. For this reason we again reply negatively to the question of the program and we try again with $D = 75\,\text{Å}$.

```
Is the experimental data fit satisfactory and the profile
correlation function approaching smoothly the z-axis near
the maximum layer extension?  [1 (yes),0 (no)]...........  0
Layer extension (Angstrom)..........................  75
```

```
Inverse Fourier transform of experimental curve
Calculating........................................
Found log(lambda) = 7.0
Inspect stability plot and suggest log(lambda) value..
```

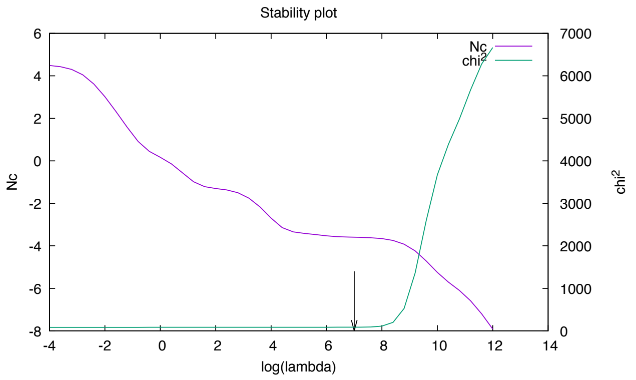

The corresponding stability plot pops-up and the found $log(\Lambda)$ value is equal to 7.

* As can be seen in the profile correlation function $p(z)$ and data fit plots, for $D = 75\,\text{Å}$, $p(z)$ is approaching smoothly the z-axis at $z = D$ while the data fit is satisfactory. That means that we have found D and we may reply affirmatively to the question of the program.

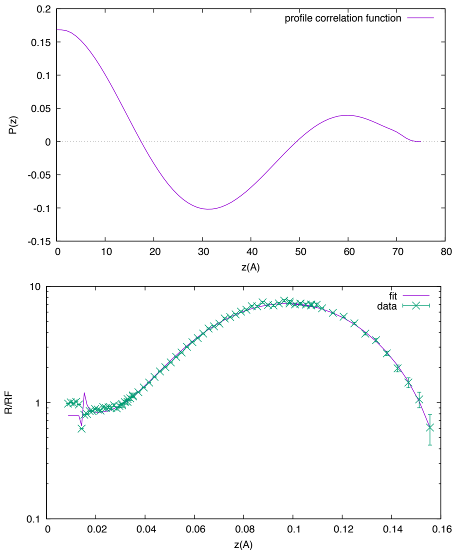


```
Is the experimental data fit satisfactory and the profile
correlation function approaching smoothly the z-axis near
```

```
the maximum layer extension?  [1 (yes),0 (no)]...........  1
```

* We continue with data input for the second contrast (H_2O).

```
Reflectivity data file name.........................  H2O.dat
Solvent sld (10^-6/Angstrom^2).......................  -0.56
```

* Now the program asks if D is known. Since we have performed IFT calculations for the D_2O contrast, we could reply 'yes' and use the previously found value. However, as a cross check and since lipid heads are expected to have a better contrast in H_2O, we may perform IFT calculations also for this dataset.

```
Is maximum layer extension known?  [1 (yes),0 (no)].....0
Layer extension (Angstrom)..........................  75
Inverse Fourier tranform of experimental curve
Calculating................................
Found log(lambda) = 7.0
```

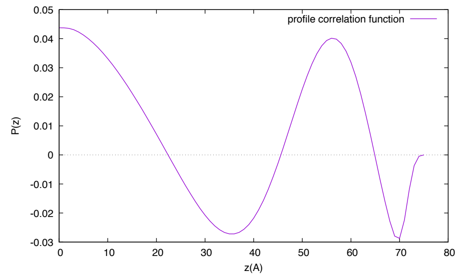

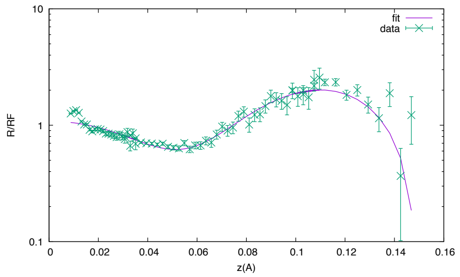

We find from the stability plot that $log(\Lambda = 7)$ and we proceed with the visualisation of $p(z)$ and the data fit.

* We can observe that the data fit is satisfactory, however it seems that $p(z)$ is not approaching the z-axis quite smoothly and maybe a slightly larger value of D might be closer to the actual value. For this reason we try with $D = 80$ Å

```
Inspect stability plot and suggest log(lambda) value..
Is the experimental data fit satisfactory and the profile
correlation function approaching smoothly the z-axis near
the maximum layer extension?  [1 (yes),0 (no)]...........  0
```

```
Layer extension (Angstrom)...........................  80
Inverse Fourier tranform of experimental curve
Calculating.......................................
Found log(lambda) = 7.4
```

* With the new IFT calculations we obtain a $p(z)$ that is approaching smoothly the z-axis at $z = D$.

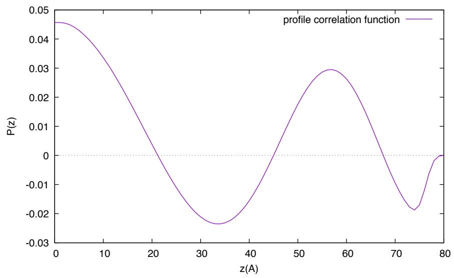

We accept this new value for D. Note that the program always uses the global maximum of D that is found by performing IFT calculations for different contrasts.

```
Is the experimental data fit satisfactory and the profile
correlation function approaching smoothly the z-axis near
the maximum layer extension?  [1 (yes),0 (no)]...........  1
```

* We then proceed with the rest of the parameters. Since no deuterated lipids were used in the experiment, we expect that the maximum sld in the system will be the one of D_2O, so we use the default value by pressing return

```
Maximum sld (10^-6/Angstrom^2) [default=6.35]........
```

* Also we expect that the sld of H_2O will be the minimum sld of the system, so again we choose the default value by pressing return

```
Minimum sld (10^-6/Angstrom^2) [default=-0.56].......
```

* For the three following input parameters we also choose the default values which are calculated based on the availiable $q-$range and the found maximum layer extension D .

```
Max smoothing parameter (Angstrom) [default= 10.1 ]..
Min smoothing parameter (Angstrom) [default= 5.0 ]..
Slab size (Angstrom) [default= 2.16]..............
```

Note: If after the end of the interfacial layer reconstruction the profiles seem unphysically smoothed, one may try to reduce the min smoothing parameter and execute a new run. By default the program is binning the profile using 40-layers. If the resulting slab thickness is not satisfactory, the user may modify the value, although the default value is expected to work for most cases.

∗ We are then asked about if we want to apply any constraints. Since we know that on the surface of silicon there exists a thin (10 Å) silica (sld=3.5 $\times 10^{-6}$ Å^{-2}) layer, we can apply the following two constraints.

```
Number of applied constraints [default=0]............  2

Input for constraint number:  1

Constraint starts at (distance from substrate in Angstrom)..  0
Constraint ends at (distance from substrate in Angstrom)....  10
Constraint type (0) hydration, (1) sld.....................  0
Hydration value (0->1)......................................  0

Input for constraint number:  2

Constraint starts at (distance from substrate in Angstrom)..  0
Constraint ends at (distance from substrate in Angstrom)....  10
Constraint type (0) hydration, (1) sld.....................  1
sld (10^-6/Angstrom^2)......................................  3.5
```

∗ For all other parameters that are related with the simulated annealing procedure we choose the default values, except from the last one that concerns the number of runs that will be performed.

```
Initial annealing temperature [default=1.00].........
Annealing schedule [default=0.90]....................
Trials per slab at each annealing step [default=100]..
Single (0) or multiple runs (1) [default multiple]....  0
```

After diplaying a summary of input parameters, the initial score function (**ScF**), is calculated and the annealing procedure then starts. Each time that the annealing temperature is lowered, the main annealing parameters (annealing temperature **T**, score function (**ScF**), smoothing parameter (**SmP**) and **suceess/trials** ratio) are printed and also output files are written on disk.

```
Initial ScF: 0.271E+01
Simulated annealing run started
T=0.900E+00| ScF=0.243E+01| SmP(Angstrom)= 7.5| success/trials= 95.6%
T=0.810E+00| ScF=0.205E+01| SmP(Angstrom)= 8.0| success/trials= 94.6%
```

```
T=0.729E+00| ScF=0.229E+01| SmP(Angstrom)= 6.0| success/trials= 94.3%
T=0.656E+00| ScF=0.260E+01| SmP(Angstrom)= 5.6| success/trials= 93.7%
```

Note that as the annealing temperature is falling, the success/trials ratio and the score function are also reduced. In rare cases where the success/trials ratio is smaller than 80% at the beginning of the annealing procedure, one has to consider to increase the initial annealing temperature.

During the simulated annealing run, the state of sld profiles, hydration profiles and the overall fit can be checked by opening the associated `.pdf` or `.dat` files.

At low annealing temperatures, and when the score function cannot be improved anymore, the program stops and reports the final achieved χ.

```
T=0.104E-03| ScF=0.295E+00| SmP(Angstrom)= 5.1| success/trials= 5.9%
T=0.940E-04| ScF=0.295E+00| SmP(Angstrom)= 5.1| success/trials= 5.9%

Chi of the model:  30.622

Elapsed time :  32.2 min
```

When multiple runs are executed, at the end the program reports the run with the lowest attained χ.

After the end of the run, we may inspect the results (fits and profiles).

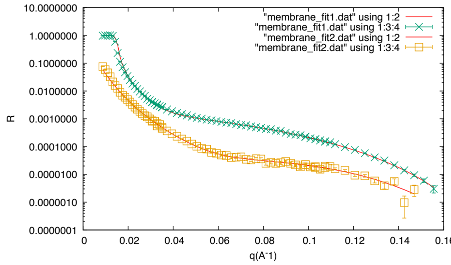

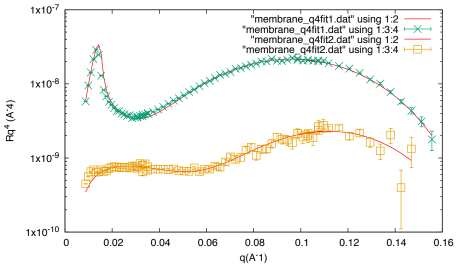

In this particular case one may see that a characteristic hydration (solvent volume fraction) and sld profile set from a supported lipid membrane system is obtained.

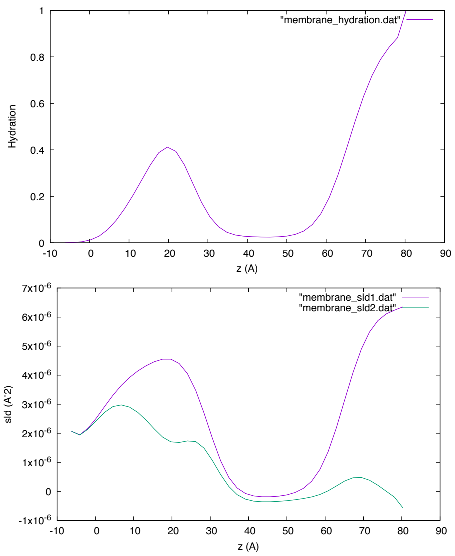

In general it is advised to run multiple annealing runs for a given dataset. Despite the longer computation time required, we may avoid cases where the annealing procedure gets stuck in local minima.

In the case where the maximum layer extension D was known beforehand, the program could have been initiated in command line argument mode using an ASCII file (see below) with the following contents.

```
# Project name
membrane
# Instrumental resolution (dq/q)
0.1
# Substrate sld (10^-6/Angstrom^2) [default=2.07]
2.07
# Number of solvent contrasts
3
# Reflectivity data file name (first contrast)
D2O.dat
# Solvent sld (10^-6/Angstrom^2) (first contrast)
6.35
# Layer extension (Angstrom) (first contrast)
75
# Reflectivity data file name (second contrast)
H2O.dat
# Solvent sld (10^-6/Angstrom^2) (second contrast)
-0.56
# Layer extension (Angstrom) (second contrast)
80
```

```
# Maximum sld (10^-6/Angstrom^2)
6.35
# Minimum sld (10^-6/Angstrom^2)
-0.56
# Maximum smoothing parameter (Angstrom)

# Minimum smoothing parameter (Angstrom)

# Slab size (Angstrom)

# Number of applied constraints
2
# 1st constraint starts at (distance from substrate in Angstrom)
0
# 1st constraint ends at (distance from substrate in Angstrom)
10
# 1st constraint type (0) hydration, (1) sld
0
# 1st constraint value
0 # 2nd constraint starts at (distance from substrate in Angstrom)
0
# 2nd constraint ends at (distance from substrate in Angstrom)
10
# 2nd constraint type (0) hydration, (1) sld
1
# 2nd constraint value
3.5
# Initial annealing temperature [default=1]
1
# Annealing schedule [default=0.90]
0.90
# Trials per slab at each annealing step [default=100]
100
# Single (0) or multiple runs (1) [default multiple]
0
```

the ASCII input file has always the format, one line 'comment' and then next line parameter.

5 Additional info

For questions related to the program, bug reports and feature requests please contact: a.koutsioumpas[at]fz-juelich.de.
